# Editorial for the Special Issue “MEMS Packaging Technologies and 3D Integration”

**DOI:** 10.3390/mi13050749

**Published:** 2022-05-09

**Authors:** Seonho Seok

**Affiliations:** Center for Nanoscience and Nanotechnology (C2N), University-Paris-Saclay, 91400 Orsay, France; seonho.seok@u-psud.fr

As fabrication technologies advance, the packaging of MEMS device is being developed in two main directions: MEMS device packaging and MEMS or sensor system integration. MEMS device packaging is an essential technique for successful commercialization of MEMS product, as MEMS devices inevitably have moving and fragile parts. MEMS devices tend to be packaged in chip-scale, or, namely, zero-level packaging as fabrication technologies advance in place of conventional package of a commercial product such as DIP (Dual Inline Package). For certain MEMS products, vacuum packaging has been achieved with zero-level packaging approaches [1,2]. Such a MEMS packaging has mainly been realized by bonding techniques with joint materials such as metals, polymers, etc. A packaging cap with housing cavity is bonded to a sealing ring surrounding MEMS devices. The lid wafers are generally fabricated with so-called hard materials such as silicon, glass, and so on. For certain MEMS devices, different cap materials, for example, thin films or polymer films, have been adopted for high frequency devices and inertial sensors [3,4,5,6,7]. Thin film packaging is fabricated by conventional semiconductor process and sacrificial etch and thus may have advantages of small package sizes and low costs due to its high throughput. The bonding techniques typically used for MEMS device packaging are interfacial bonding and intermediate layer bonding [8,9,10,11,12]. The interfacial bonding depends on the chemical reaction between two joint materials, while the intermediate layer bonding needs additional materials as adhesive layers. Anodic bonding and silicon fusion bonding are frequently used interface bonding techniques. The interfacial bonding requires high surface cleanness as well as high surface flatness, and it is carried out under high temperature and high applying pressure conditions. Thus, the interfacial bonding has certain constraints for temperature sensitive MEMS devices. Intermediate layer bonding needs good adhesion materials with associated substrates to avoid unwanted delamination of the sealing layers. As the intermediate layer material determines the bonding condition such as temperature, it can be implemented at relatively low temperature. For the packaging based on bonding technologies, attention should be paid to thermal expansion coefficient difference among the associated materials because it would cause undesired high packaging stress. Packaging stress is a principal cause of its reliability, and thus modeling and simulation of electronic and MEMS packages have been frequently performed through FEM (Finite Element Method) to understand mechanical behavior of the packages [13,14,15,16,17]. To obtain reliable simulation results, material properties and their behavior depending on temperature or external load should be well characterized. As an alternative packaging approach, thin film encapsulation integrates the packaging process with the MEMS device process on the same wafer. MEMS structures covered by an additional sacrificial layer are first released by sacrificial etching through channels or holes, and then the access holes are sealed by depositing an overcoat material. The thin-film packaging materials should be deposited or formed without degrading or changing the properties of MEMS structure, and it takes a longer time to release overall packaging cap including the packaged MEMS devices via the accesses of etching solution or gas [18]. MEMS packaging has application-specific features. In other words, it has different approaches depending on application. For example, high-performance inertial sensors may need vacuum packaging fabricated with solid metal-to-metal bonding, but it could not be applied to RF-MEMS packaging because such a metallic sealing ring may create additional loss or undesired parasitic effect at higher frequencies. Another aspect of MEMS product packaging is integration and packaging with integrated circuit (IC) chips, as MEMS output signals should be processed for operation. The integration of MEMS and ICs is implemented through a hybrid multi-chip solution or an SoC solution. SoC solutions have the fabrication and integration of MEMS and IC components implemented on the same substrate, and the fabricated chips are separated only at or near the end of the fabrication process. The hybrid integration of MEMS and IC technology has been implemented by 2D integration approaches. MEMS and IC wafers are fabricated independently and placed on a common printed circuit board (PCB) and then interconnected with wire-bonding. Multi-Chip-Module (MCM) is an advanced integration technique of hybrid integration; MEMS and IC chips are placed side-by-side in a common package and interconnected at the package level, typically via wire and/or flip-chip bonding [19]. This approach has evolved into system-in-packages, also referred to as vertical or stacked multi-chip modules, consisting of chips that are attached on top of each other and interconnected via wire and/or flip-chip bonding, either directly or through additional re-distribution layers. The main benefits of these 3D stacked approaches are their higher integration densities, shorter signal path lengths, and smaller package footprints/volumes in comparison with the 2D multi-chip modules. Moreover, package-level system-on-packaging can integrate optics, wireless communication, and power module along with MEMS and IC on a common package. For the packaging and integration explained earlier, interconnection techniques have been important as the packaging size determines the cost of the final product [20,21,22]. Interconnect techniques frequently used for MCM packaging are wire-bonding, solder balls, metal stud bumps, and ACF (Anisotropic Conductive Film). Most of the interconnected techniques require certain amount of pressure at elevated temperature for efficient bonding between materials in joint. In case of metallic joints, such as solder balls and metal stud bumps, a relatively thin adhesion or UBM (under bump metallurgy) layer would be critical for the package reliability, as the adhesion layer could be delaminated or disconnected due to intermetallic diffusion. In certain cases, the length of metallic interconnect determines the life-time of the package due to shear stress limit [23].

Due to the emergence of novel electronic devices such as flexible electronics or implantable medical devices, the Si IC should be integrated with flexible substrate to comply with new applications. Two-dimensional-material-based circuit approaches are attractive for flexible electronics, but advances in key areas such as robust manufacturing and reliable mechanical characterization are still necessary [24]. The integration of existing IC and novel biocompatible polymeric devices is highly demanded for new implantable medical devices, for example, neural prosthetics [25,26]. Such medical devices should be encapsulated in a biocompatible way for human body implantation. The reliability and life-time of the implant system are highly dependent on both packaging materials and technology. In general, implantable device packaging houses the electronic or mechanical system through the polymer encapsulation [27], welding, or bonding of metal [28,29] and ceramics [30]. Materials of the polymer encapsulation package include epoxies, silicones, polyurethanes, polyimides, silicon-polyimides, parylenes, polycyclic-olefins, silicon-carbons, benzocyclobutenes (BCB), and liquid crystal polymers. Furthermore, the packaging size of the implant should be minimized to avoid unwanted foreign body reaction (FBR) during implantation. The biocompatible packaging has been shown in different implantable medical devices, for example, a cardiac monitoring system implemented with commercial three-axis accelerometer, pressure sensor device mounted on a stent graft, implantable retina stimulator implemented by MFI (MicroFlex Interconnection) technology, thin-film interconnect for 1000-electrode retina prothesis, etc. [31].

In conclusion, MEMS packaging has been evolved from MEMS device packaging to MEMS system packaging as the application of MEMS devices has been widely extended. Innovative and efficient packaging technologies becomes more and more important as well as new packaging materials. Concerning heterogeneous integration highly demanded for new applications, the interconnection between different material such as silicon and polymers should be adapted in order to reduce mechanical and electrical optimization. This Special Issue presents 12 research papers and 1 review article on recently developed MEMS packaging technologies and 3D integration. It will serve to elucidate the need for new packaging technologies and its recent research trend.
